# Availability and accessibility of subsidized mammogram screening program in peninsular Malaysia: A preliminary study using travel impedance approach

**DOI:** 10.1371/journal.pone.0191764

**Published:** 2018-02-01

**Authors:** Aidalina Mahmud, Syed Mohamed Aljunid

**Affiliations:** 1 International Centre for Casemix and Clinical Coding, Faculty of Medicine, Universiti Kebangsaan Malaysia, Kuala Lumpur, Malaysia; 2 Department of Health Policy & Management, Faculty of Public Health, Health Science Centre, Kuwait University, Kuwait; University of Zurich, SWITZERLAND

## Abstract

Access to healthcare is essential in the pursuit of universal health coverage. Components of access are availability, accessibility (spatial and non-spatial), affordability and acceptability. Measuring spatial accessibility is common approach to evaluating access to health care. This study aimed to determine the availability and spatial accessibility of subsidised mammogram screening in Peninsular Malaysia. Availability was determined from the number and distribution of facilities. Spatial accessibility was determined using the travel impedance approach to represent the revealed access as opposed to potential access measured by other spatial measurement methods. The driving distance of return trips from the respondent’s residence to the facilities was determined using a mapping application. The travel expenditure was estimated by multiplying the total travel distance by a standardised travel allowance rate, plus parking fees. Respondents in this study were 344 breast cancer patients who received treatment at 4 referral hospitals between 2015 and 2016. In terms of availability, there were at least 6 major entities which provided subsidised mammogram programs. Facilities with mammogram involved with these programs were located more densely in the central and west coast region of the Peninsula. The ratio of mammogram facility to the target population of women aged 40–74 years ranged between 1: 10,000 and 1:80,000. In terms of accessibility, of the 3.6% of the respondents had undergone mammogram screening, their mean travel distance was 53.4 km (SD = 34.5, range 8–112 km) and the mean travel expenditure was RM 38.97 (SD = 24.00, range RM7.60–78.40). Among those who did not go for mammogram screening, the estimated travel distance and expenditure had a skewed distribution with median travel distance of 22.0 km (IQR 12.0, 42.0, range 2.0–340.0) and the median travel cost of RM 17.40 (IQR 10.40, 30.00, range 3.40–240.00). Higher travel impedance was noted among those who lived in sub-urban and rural areas. In summary, availability of mammogram facilities was good in the central and west coast of the peninsula. The overall provider-to-population ratio was lower than recommended. Based on the travel impedance approach used, accessibility to subsidised mammogram screening among the respondents was good in urban areas but deprived in other areas. This study was a preliminary study with limitations. Nonetheless, the evidence suggests that actions have to be taken to improve the accessibility to opportunistic mammogram screening in Malaysia in pursuit of universal health coverage.

## Introduction

Early diagnosis of breast cancer by mammography screening has become a critical way to reduce mortality [1]. Breast cancer survival drops dramatically for late stage diagnoses [2]. In the randomized controlled trials for women aged 40 to 74 years, screening with mammography has been associated with a 15% to 20% relative reduction in mortality from breast cancer [3]. A prospective cohort study in Norway found that invitation to modern mammography screening may reduce deaths from breast cancer by about 28% [4].

Women without adequate accessibility to timely mammography screening are more likely to develop late-stage breast cancer [5]. A study showed that advanced diagnoses had longer average travel distances than early stage diagnoses. After adjusting for age, race, insurance and education, the odds of advanced diagnosis were significantly greater for women residing over 15 miles from a facility, compared to those living within 5 miles [6].

Breast cancer screening program can either be an organized or opportunistic program. In an organized program, invitations are issued from centralized population registers. In opportunistic screening program, attendance to screening depend on the individual’s decision or on encounters with health care providers where screening may be recommended. Although both approaches may yield similar uptake rates, organized programs are more likely to reduce breast cancer mortality because of the use of central registers for invitation, and the centralized commitment to quality assurance, monitoring, and evaluation [7].

Breast cancer is the most common cancer in Malaysia, accounted for 32.1% of all female cases. The age-standardized rate (ASR) was highest in the 50–59 age group. The incidence of breast cancer was highest among the Chinese ethnicity with ASR of 41.5 per 100,000 population, Indian (ASR of 37.1 per 100,000 population) and Malay (ASR 27.2 per 100,000 population). The percentage of breast cancer detected at stage I was 20%, stage II 37%, Stage III 23% and stage IV 20% [8].

The Malaysian Clinical Practice Guideline on the Management of Breast Cancer, 2010 [9] recommends the following: mammography may be performed biennially in women from 50–74 years of age; breast cancer screening using mammography in low and intermediate risk women aged 40–49 years old should not be offered routinely but these women should not be denied mammography screening if they desire to do so; breast self-examination is recommended for raising awareness among women at risk rather than as a screening method; screening women at high risk for breast cancer should be done from the age of 30 years with both MRI and mammography as they are more effective than mammography alone and MRI screening should not be performed in patients with lobular carcinoma in situ and atypical hyperplasia [9].

In Malaysia, breast cancer screening by means of mammography is part of an opportunistic screening program. In general, breast self-awareness is promoted among women. However, based on the National Guideline for the Early Detection of Breast Cancer Program 2011 [10], if a woman was found to have high risk for breast cancer, she will be eligible for mammogram screening. The mammogram screening can be done at government hospitals with mammogram facility; or at private hospitals appointed by the National Population and Family Development Board (NPFDB) Malaysia mammogram screening program. NPFDB is an entity under the Ministry of Women, Family and Community Development which has carried out a subsidized mammogram program since the year 2007. With this program, women can either undergo mammogram screening for free or with a minimal fee of RM50.00 depending on their income. Other entities which provide mammogram screening subsidy include the Social Security Organization (SOCSO) and several State Governments. Alternatively, a woman may undergo mammogram at private healthcare facilities and pay out-of-pocket for it. A mammogram investigation may cost RM230 or more based on the Private Healthcare Facilities and Services (Private Hospitals and Other Private Healthcare Facilities) (Amendment) Order 2013 [11] and this amount approximates to about 4.0% of median monthly income for urban population (RM 5, 156) or 7.0% of median monthly income for rural population (RM 3, 123) [12]. This cost does not include the registration fee, doctor’s consultation fees and travel costs.

Despite the availability of subsidized screening programs, a review of thirteen local studies published between the years 2006 and 2014 indicated that the rate of mammogram uptake in the country ranged between only 6.8% and 80.3% [13–23] According to the studies conducted in the urban and sub-urban localities of Terengganu, Selangor and Kuala Lumpur, mammogram screening uptake was between 10.5% and 31.9% among the general population and was 80.3% among hospital staff [24]. However, mammogram screening uptake was noted to be less than 10% (6.8% to 8.3%) among women in rural localities of the states of Perak and Pahang. However, none of these studies had addressed the accessibility of the subsidized mammogram screening program.

Accessibility to services is one of the many ways to achieve universal health coverage (UHC). Access is a multidimensional concept, based on five main dimensions—affordability, accommodation, acceptability, availability and accessibility [25]. Access can be divided into spatial and non-spatial. Affordability, accommodation, acceptability are non-spatial in nature—they address health care financing arrangements and access barriers created by socio-economic and cultural factors. On the other hand, availability and accessibility are spatial in nature. Availability address the adequacy of the supply of health care providers, while accessibility, namely spatial accessibility, addresses travel barriers (travel distance, cost and duration) to health care providers.

Measuring spatial accessibility is common approach to evaluating access to health care [26–28]. Methods to measure spatial accessibility to health care can be broadly divided into two [29]: revealed accessibility methods and potential accessibility methods. Revealed accessibility refers to methods that use data from actual healthcare trips, for example the drive time or straight-line distance between a patient’s home address and the hospital they attended [30, 31]. Potential accessibility refers to methods that look at what is the potential for accessing healthcare facilities in a particular area, for example, using gravity models originally developed by Hansen in 1959 [32] and specialised gravity models—such as, two-step flotation catchment area method [33,34]. Travel impedance to nearest provider method is one of the methods used to estimate revealed accessibility. Travel impedance is typically measured from a patient’s residence, often in units of Euclidean (straight line) distance and may include travel cost and travel time. This travel impedance method was used in this current study because it could provide the most suitable data in terms of travel distance and more importantly travel expenditure. By virtue of their point-to-point nature, travel impedance measures have an advantage over the other methods measuring accessibility, as they are able to account for border-crossing behaviours [35,36] and hence it was particularly appropriate for rural areas, where provider choices are limited and the nearest provider is usually the one most likely to be utilized [37].

The main objective of this study was to determine the availability and spatial accessibility of subsidised mammogram service in Malaysia. The specific objectives were to determine the availability of mammogram screening program providers, the locations of facilities used in these programs, the estimated travel distance and the estimated travel expenditure incurred by the women undergoing the subsidized mammogram.

## Methods

This study is part of a larger study on assessing the extent of universal health coverage in breast cancer management in Malaysia. Approval for the research was obtained from the Universiti Kebangsaan Malaysia Research Ethics Committee and the National Medical Research Registry Ethics Committee. This study comprised of two sections. The first section involved collecting information on the available subsidised mammogram screening programs, the number of women aged 40–74 years and the current locations of facilities involved in mammogram screening programs, up to the year 2015. Data on the estimated target population for the year 2015 was extracted from the Department of Statistics Malaysia, Official Portal, while the list of facilities involved in subsidized mammogram screening was compiled from the lists available at the websites of subisdized mammogram program providers. From this data, availability of subsidised mammogram services in terms of the provider-to-population ratio was determined. Also, addresses of the facilities were entered into a mapping application to map the locations and distribution of these facilities.

The second section involved estimating the travel impedance (i.e. travel distance and travel expenditure) experienced by the respondents. This was done through a cross-sectional study among breast cancer patients who received treatment in four referral tertiary level hospitals with surgical, oncology and radiation therapy services (Hospital Kuala Lumpur, Hospital Putrajaya, National Cancer Institute and Universiti Kebangsaan Malaysia Medical Centre) in the years 2015–2016. These hospitals were located in the central region of Malaysia, where they serve not only population in their locality but also patients referred from other parts of the country. Breast cancer patients were recruited via purposive sampling. Criteria for eligibility were—Malaysian female, able to communicate in the national language or English, diagnosed with breast cancer as the primary oncology diagnosis and clinically stable (comfortable and able to communicate). The respondents who were approached were informed about the study, the study objectives and what was required from the respondents. After obtaining informed consent from the respondents, data was collected through questionnaire-guided interview. The respondents were asked about their age, ethnicity, occupation, stage of breast cancer at diagnosis, where they lived (locality and district) and the locations of the mammogram facility if they had undergone mammogram screening.

In this study, the estimation of total travel distance was *the sum* of travel distance to each facility the respondent went to, multiplied by two to account for the return trips made. This travel distance was determined based on the respondent’s housing area or locality and the nearest assessment clinic and facility with mammogram, using a mapping application Although there is no formal threshold of the accepted travel distance for health care, several studies have used the threshold for high travel burden as 30 miles (48.3 kilometres) [37,38]. Anyone having to travel more than this distance was regarded as having high travel burden. Travel time was not included because it does not represent burden travel in Malaysia. Longer travel time would not necessarily mean shorter travel distance because in the urban areas, travel time may take longer due to traffic congestion.

Cost analysis of out-of-pocket expenditure for travel to and from the facilities and parking fees incurred in the subsidized mammogram screening was carried out. The cost per kilometre travelled was set at RM 0.70 which was the rate for government staff travel allowance using privately-owned cars] [39]. This method of calculation was also used in another similar study [40]. All costs are presented in 2015 Malaysian Ringgit (RM).

## Results

### Availability

Malaysia consists of thirteen states and three federal territories with a total landmass of 330,803 square kilometres. There are two similarly sized regions, Peninsular Malaysia and East Malaysia, separated by the South China Sea. In this study the Peninsula Malaysia was divided into several regions: central and west coast region (Kuala Lumpur, Selangor, Negeri Sembilan), southern region (Melaka and Johor), northern region (Perak, Kedah, Pulau Pinang (Penang), Perlis) and eastern region (Pahang, Terengganu and Kelantan).

As of the year 2015, several major entities offered subsidised mammogram screening in Malaysia. These entities were the Ministry of Health, the National Population and Family Development Board Malaysia (NPFDB); the Social Security Organization (SOCSO) and two state governments. The National Cancer Council Malaysia (NCCM) also provide free mammogram services using one mobile mammogram machine housed in a 40-feet mobile trailer. Except for Ministry of Health and NCCM’s mobile screening unit, all the other entities do not have their own facilities with mammogram—they only provide the funding for mammogram and the service was outsourced to private facilities (clinics, diagnostic centres and hospitals).

The density of mammogram facilities was estimated based on the number of the target population (women between the ages 40–74 years old for the year 2015), and the number of facilities involved with the subsidized mammogram screening program available. In the central and west coast regions, the ratio mammogram facility to the target population was about 1:10,000–20,000; while in other parts of the country it was approximately 1:20,000–1: 80,000. The ratio of facilities involved in providing mammogram to target population according to state is detailed in Table 1.

**Table 1:** Ratio of facilities involved in providing mammogram to target population according to state.

State	Total facilities providing subsidised mammogram (n)	Target population (women aged 40-74y)	Ratio facility: target population
KL and Putrajaya	25	252,800	1: 10,112
Selangor	37	749,000	1: 20,243
Perlis	1	36,900	1: 36,900
Pulau Pinang	12	271,000	1: 22,583
Kedah	8	315,200	1: 39,400
Perak	11	400,300	1: 36,390
Negeri Sembilan	9	160,900	1: 17,877
Melaka	4	131,900	1: 32,975
Johor	17	484,700	1: 28,511
Pahang	6	207,200	1:34,616
Terengganu	2	145,700	1: 72,850
Kelantan	3	237,100	1: 79,033
Sabah & Labuan	11	309,500	1: 28,136
Sarawak	14	366,700	1: 26,192

In order to undergo subsidized mammogram screening, a woman may need to visit firstly the assessment clinic, then the facility with mammogram [41–44]. At the assessment clinic, the woman would undergo registration, breast cancer risk assessment, clinical breast examination and be given health education on breast awareness. If abnormality was detected the woman would be referred to the hospital for further management. Otherwise she would be given an appointment date for mammogram. In some instances, the woman may need to go or contact the facility of choice to make an appointment. After the woman had undergone the procedure, she may be given the report. The interpretation of the report may be done at the mammogram facility itself or at the initial assessment clinic. If the result was abnormal, the woman was required to undergo further investigations, but if the result was normal, she would be given a date for the next mammogram. These steps were simplified when, in 2013, the National Population and Family Development Board Malaysia (NPFDB) appointed 50 one-stop mammogram facilities called entry points where a woman may directly get the mammogram without having to attend the assessment clinic beforehand.

### Accessibility

A total of 364 breast cancer patients who were receiving treatment at the selected hospitals were approached. Of these, thirteen did not meet the inclusion criteria while seven refused to participate. Among the seven non-respondents, two were of Malay ethnicity, two Chinese and three Indian. The remaining number of patients who fulfilled the inclusion criteria and agreed to participate were 344. The age of these respondents was categorized into two groups: less than 40 years and 40 years and more. The age of 40 years was used as a cut-off point based on the eligibility criteria of the available mammogram screening programs. Of the total number of respondents, 48 were diagnosed with breast cancer before the age of 40 years and thus were not in the age group for mammogram screening. They were then excluded from the subsequent analysis. The sociodemographic characteristics of the remaining 296 respondents are in Table 2.

**Table 2:** Sociodemographic characteristics of respondents aged 40 years and more (n = 296).

Sociodemographic variable	Frequency (Percentage)
**Age**	
Mean age (± SD)	54.1 (8.1) years
**Ethnicity**	
Malay	178 (60.1%)
Chinese	71 (24.0%)
Indian	47 (15.9%)
**Occupation (sector)**	
Government/pensioner	64 (21.6%)
Private/ self-employed	53 (17.9%)
Not employed/housewife	179 (26.7%)
**Marital status**	
Married	216 (73.0%)
Divorced	14 (4.7%)
Widowed	44 (14.9%)
Single	22 (7.4%)

Among these respondents, only eleven (3.6%) claimed to have undergone mammogram screening on their own accord prior to being diagnosed with breast cancer. The frequency of mammogram screening done, time lapse between the mammogram screening and diagnosis; or the respondents’ perception on mammogram screening were not ascertained as these constituted another aspect of access (i.e. acceptability) which was beyond the scope of this study. The stage and status of mammogram screening for the respondents aged 40 years and more are summarized in Table 3.

**Table 3:** Stage and status of mammogram screening for respondents aged ≥ 40 years.

Stage at diagnosis	Number of respondents aged ≥ 40 years	Did mammogram screening?	
		**Yes**	**No**
≤ I	7	1	6
II	84	2	82
III	113	7	106
IV	92	1	91
Total	296	11	285

For the respondents aged 40 years and above who had undergone mammogram screening, the travel distance and travel expenditure were calculated. The findings are summarized in Table 4.

**Table 4:** Characteristics of the respondents, travel distance and travel expenditure.

(a)	(b)	(c)	(d)	(e)	(f)	(g)	(h)	(i)	(j)	(k)	(l)	(m)	(n)	(o)	(p)
Respondent	Age (years)	Marital status	Occupation	Race	State/zone	Distance (home to assessment clinic)(km)	Number of trips (home to assessment clinic)	Total travel distance (home to assessment clinic) [g*h*2] (km)	Distance (home to facility with MMG)(km)	Number of trips (home to facility with MMG)	Total travel distance (home to facility with MMG) (km) [k*l*2]	Overall total travel distance (km) [i+l]	Total travel expenses (RM) = m * RM 0.70	Total parking fee (RM)	Grand total travel expenditure (RM) = [n + o]
A	54	married	retiree	Malay	Negeri Sembilan	0	0	0	4	1	8	8	5.60	2.00	7.60
B	46	married	housewife	Malay	Selangor	2	2	8	32	1	64	72	50.40	0.00	50.40
C	55	married	teacher	Indian	Selangor	0	0	0	35	1	70	70	49.00	2.50	51.50
D	52	married	teacher	Malay	Selangor	5	2	20	25	1	50	70	49.00	2.00	51.00
K	58	married	clerk	Indian	Selangor	2	2	8	52	1	104	112	78.40	0.00	78.40
E	48	married	housewife	Malay	Kuala Lumpur	0	0	0	7	1	14	14	9.80	0.00	9.80
F	55	married	housewife	Chinese	Kuala Lumpur	0	0	0	8	1	16	16	11.20	2.00	13.20
G	42	married	housewife	Malay	Johor	7	2	28	29	1	58	86	60.20	2.00	62.20
H	52	married	housewife	Malay	Kuala Lumpur	0	0	0	15	1	30	30	21.00	2.50	23.50
I	58	married	retiree	Indian	Selangor	0	0	0	38	1	76	76	53.20	3.00	56.20
J	56	divorced	self -employed	Chinese	Kuala Lumpur	0	0	0	16	1	32	32	22.40	2.50	24.90

The total travel distance experienced by the respondents who underwent mammogram ranged between 8 km and 112 km, with a mean of 53.4 km (SD 34.5). Of these, five respondents (45%) had travel distance less than 48 km. As all these respondents had stated that they used personal car, the distance travelled was multiplied by RM 0.70. The total travel expenditure inclusive of the parking fee ranged between RM 7.60 and RM 78.40, with a mean of RM 38.97 (SD 24.00).

For respondents aged 40 years and above who *did not* go for mammogram screening, the travel distance and travel expenditure were also estimated. In these estimations, it was assumed that these respondents utilized personal vehicle (car) instead of public transportation and they had gone to the nearest facility with subsidized mammogram screening. If this nearest facility was not an entry-point facility then it was assumed that the respondent went to the nearest assessment clinic before going to the mammogram facility. Parking fees was estimated using the rate of RM2.00 per trip. This rate was based on the average hourly parking rates of major private hospitals in Malaysia.

Among the respondents who *did not* undergo mammogram screening, their sociodemographic characteristics are as shown in Table 5.

**Table 5:** Sociodemographic characteristics of respondents who did not undergo mammogram screening (n = 285).

Sociodemographic variable	Frequency (Percentage)
**Age**	
Mean age (± SD)	54.2 (8.2) years
**Ethnicity**	
Malay	171 (60.0%)
Chinese	69 (24.2%)
Indian	44 (15.4%)
**Occupation (sector)**	
Government/pensioner	60 (21.0%)
Private/ self-employed	51 (17.9%)
Not employed/housewife	174 (61.1%)
**Marital Status**	
Married	206 (72.3%)
Divorced	13 (1.1%)
Widowed	44 (15.4%)
Single	22 (7.7%)

If these respondents had gone for mammogram screening, those who stayed in the central and west regions of the country would have shorter travel distance compared to those in the other states, as shown in Table 6.

**Table 6:** Travel distance for the respondents if they had gone for mammogram screening.

Total travel distance (km)	Location of patients’ residences	Total (%)
<10	Kuala Lumpur, Selangor, Melaka, Negeri Sembilan, Johor,	42 (14.7%)
10–< 20	Kuala Lumpur, Selangor, Negeri Sembilan, Perak	74 (26.0%)
20–< 30	Kuala Lumpur, Selangor, Melaka, Pahang	57 (20.0%)
30–< 40	Selangor, Negeri Sembilan, Johor, Perak, Kuala Lumpur	35 (12.3%)
40–< 50	Selangor, Kedah, Kuala Lumpur, Negeri Sembilan, Perak, Johor	17 (6.0%)
50–< 60	Selangor, Negeri Sembilan, Kuala Lumpur	8 (2.8%)
60–< 70	Selangor, Perak, Kuala Lumpur	10 (3.5%)
70–< 80	Negeri Sembilan, Selangor, Johor, Melaka	9 (3.2%)
80–< 90	Negeri Sembilan, Selangor, Perak	8 (2.8%)
90–< 100	None	0 (0%)
≥100	Perak, Johor, Terengganu, Kelantan, Negeri Sembilan, Pahang, Selangor	25 (8.8%)
Total		285 (100%)

If they had gone for the mammogram screening, the total travel distance they would have experienced was estimated to range between 2 km and 340 km. The distribution of the data was skewed with a median of 22.0 km (IQR 12.0–42.0). The total travel expenditure inclusive of parking fee ranged between RM 3.40 and RM 240.00, a median of RM 17.40 (IQR 10.40–30.00). The majority of the respondents who did not undergo mammogram screening (n = 225, 79%) had travel distance of less than 48 km. The locations of respondents who had travel distance of 48 km and more are shown in Table 7.

**Table 7:** Locations of respondents who had travel distance of 48 km and more.

State	Travel distance <48 km	Travel distance ≥ 48 km
Kuala Lumpur	Sentul, Kampung Baru, Pandan Jaya Pandan Perdana, Kepong, Cheras, Bukit Desa, Jalan Ipoh, Jalan Klang Lama, Ampang, Jalan Peel, Jalan Genting Kelang, Desa Pandan, Taman Maluri, Taman Keramat, Sungai Besi, Setapak, Pantai Dalam, Jinjang, Bandar Tasik Selatan, Wangsa Maju, Ulu Kelang, Bukit Jalil, Bangsar.	**None**
Selangor	Shah Alam, Klang, Subang Jaya, Petaling Jaya, Bandar Baru Ampang, Bandar Tun Hussein Onn Cheras, Bandar Puchong Jaya, Damansara, Bangi, Kajang, Jenjarom, Batu Caves, Selayang, Sungai Buloh, Kelana Jaya, Seri Kembangan, Setia Alam, Sepang, Rawang, Tanjung Karang, Kapar, Kota Damansara, Puncak Alam, Hulu Langat.	Beranang, Section U10 Shah Alam, Sungai Besar, Tanjung Karang, Banting, Kuala Kubu Baru, Kuang.
Negeri Sembilan	Tampin, Senawang, Seremban.	Gemas, Jelebu, Lenggeng.
Melaka	Batu Berendam, Durian Tunggal, Peringgit.	Jasin
Johor	Batu Pahat, Kluang, Johor Bahru, Muar.	Kluang, Segamat, Kota Tinggi,
Perak	Ipoh, Teluk Intan, Hutan Melintang, Batu Gajah.	Sungkai, Lumut, Trolak, Slim River, Kampar, Sungai Siput, Jalan Tapah
Kedah	**None**	Sungai Petani, Kuala Ketil
Pahang	**None**	Kuantan, Bera, Temerloh
Kelantan	**None**	Tanah Merah
Terengganu	**None**	Kuala Terengganu, Bandar Al-Muktafibillah Shah.

Mann-Whitney U test was done to compare whether there were differences in the travel distance and travel expenditure between respondents who did go for mammogram screening and those who did not go. Travel distance among respondents who did go for mammogram screening was higher than those who did not go but this difference is not statistically significant (*p* = 0.065) (Tables 8 and 9). Similarly, travel expenditure among respondents who did go for mammogram screening was higher than those who did not go but this difference is not statistically significant (*p* = 0.063) (Tables 10 and 11).

**Table 8:** Mann-Whitney U mean ranks table—Travel distance by respondents.

	Ranks			
	Respondents categories	N	Mean Rank	Sum of Ranks
Travel distance of those who went for mammogram and those who did not	No Go	285	146.70	41808.50
	Go	11	195.23	2147.50
	Total	296		

**Table 9:** Mann-Whitney U test statistics—Travel distance by respondents.

Test Statistics	
	Travel distance
Mann-Whitney U	1053.500
Wilcoxon W	41808.500
Z	-1.847
Asymp.Sig. (2-tailed)	0.065

**Table 10:** Mann-Whitney U mean ranks table—Travel expenditure by respondents.

	Ranks			
	Categories	N	Mean Rank	Sum of Ranks
Travel expenditure of those who went for mammogram and those who did not	No Go	285	146.68	41804.50
	Go	11	195.59	2151.50
	Total	296		

**Table 11:** Mann-Whitney U test statistics—Travel expenditure by respondents.

Test Statistics	
	Travel expenditure
Mann-Whitney U	1049.500
Wilcoxon W	41804.500
Z	-1.862
Asymp.Sig. (2-tailed)	0.063

## Discussion

In terms of availability, there were many subsidised screening programs which catered for various groups of women. There were at least 6 major subsidised mammogram screening programs in Malaysia in 2015. The Ministry of Health and NPFDB cater for the general population, SOCSO for women in the formal employment sector, NCC for rural women and State Governments for their constituents. The number of programs in this country was more than what in the neighbouring country, Singapore. In Singapore there were three subsidized mammogram screening programs under: 1) the Health Promotion Board’s (HPB) Screen for Life [45], the Medisave and the Singapore Cancer Society (SCS) [46].

There seemed to be maldistribution of facilities with mammogram. Most of facilities with mammogram were mainly located in the central and the west coast regions of Peninsula Malaysia where major cities are located. This scenario is similar to the neighbouring countries such as Thailand—where 50% of the total mammogram facilities were concentrated in Bangkok as opposed to only 5% in the northern region of the country; and only about 30 provinces have mammogram facilities while the remaining approximately 46 provinces have none [47].

The ratio of mammogram facility to the target population was good in the central region particularly in Kuala Lumpur where the ratio was 1:10,000 (assuming one facility with mammogram had one mammogram machine). However the ratio was poor in other parts of the country, ranging from 1:20,000 to 1: 80,000. These densities are far below the threshold of the US Healthy People 2010 target screening rate which was 1.2 machines to 10,000 women aged 40 or older to achieve a target of 70% of women being screened [48]. However, it is important to note that most developed nations have population-based mammogram screening programmes, for which target screening rates are set. In Malaysia, population-based mammogram screening programme is not yet available, therefore such target screening rate would be very applicable, nonetheless this threshold gives a rough guide as to how many facilities are required.

The mean travel distance of the eleven women who underwent mammogram screening was 53.4 km (SD = 34.5, range 8–112 km) and the mean travel cost was RM 38.97 (SD = 24.00, range RM 7.60–78.40). Among respondents who *did not* go for mammogram screening, the distribution of travel distance they would have experienced if they had gone was skewed, the range was estimated to be 2–340 km; with a median of 22km (IQR 12, 42), while the estimated travel cost would have been between RM 3.40–240.00 (median RM17.40, IQR 10.40, 30.00).

Studies have shown that women who lived farther away from facilities were less likely to undergo mammogram screening than those who lived closer to facilities. An example is an American study which showed that even if mammogram screening was offered free of charge, women may not use the service if they must travel more than 20 miles (32.2 km) to receive it [49]. An Australian study suggested that a woman’s probability of attending mammogram screening declined by approximately 3% in a multiplicative manner for every additional kilometre her postcode was located from the program [50]. Another study showed that the attendance for mammogram decreased by approximately 2% for each increase in kilometre from the screening unit [51]. One study showed that after adjusting for age, race, and county education level, the odds of receiving a mammogram was slightly lower for persons residing longer distances from a permanent facility (odds ratio = 0.97) for each 5-mile (8 km) increase in distance [52]. A Canadian study showed that compared to women living <2.5 km from a screening centre, absolute decrease of 6.3% in participation rate were observed for distances of 50.0 to <75.0 km, and decrease of 9.8% in participation rate for distance ≥75.0 km [53]. A Malaysian study showed that the non-compliant patients for mammogram lived farther away (mean distance was 51.1 km) from the facilities compared to the control group (26.5km) [54].

Although these studies show that high travel burden (i.e. long travel distance) can be a barrier to undergoing mammogram screening, in this current study the majority (n = 6, 55%) of the respondents who *did undergo* mammogram screening had *high* travel burden; while the majority (n = 225, 79%) of respondents who *did not undergo* mammogram screening had *low* travel burden of less than 50km. However further analysis showed that his difference was not statistically significant. Other factors which could contribute to not going for mammogram screening apart from travel distance, need to be further investigated in future studies as they were not within the scope of this current study.

It was also noted in this study that respondents who had had travel distance of less than 48km lived in urban areas, while those who travelled 48km or more lived in mostly sub-urban and rural areas. In extremely rural areas, the travel distance was as high as 340km hence low accessibility to subsidised mammogram screening.

Recommendations for measures that can be taken to overcome issues related to availability and accessibility featured in this study include—increasing the number and distribution of facilities with mammogram especially to the rural areas. Alternatively the number of mobile mammogram screening services to rural women and housewives can be increased; and the screening process should be simplified to reduce travel distance due to multiple trips.

Recommendations for future research include a conducting a similar study in a larger scale (eg. nationwide), apply other methods of measuring (potential) spatial accessibility such as the two step floating catchment area (2SFCA) method, conduct qualitative research on the accessibility of mammogram screening to explore spatial barriers and gather suggestions by end user on how to reduce these barriers.

## Limitations

This study has several limitations. This study was conducted in tertiary referral hospital in the central region Peninsula of Malaysia, hence it did not capture respondents in other parts of the country especially of East Malaysia. Due to the sampling method used, the racial distribution of the sample did not exactly match the distribution of prevalence of breast cancer as stated in the available national cancer registry. Travel burden estimate for the respondents who did not go for mammogram screening was done based on several assumptions in terms of mode of transport (car) and choice of facility (nearest provider).

## Conclusion

This study provides preliminary findings on availability and accessibility to subsidized mammogram screening in Peninsular Malaysia using the travel impedance approach. Although there were many subsidized screening programs available in the country, these programs were dependent upon the availability of facilities with mammogram which are more densely located in the central and west coast regions and urban areas. Mammogram facility-to-population ratio was still low compared to the recommended ratio of countries which have organized mammogram screening program.

In terms of accessibility, the travel burden for the majority of respondents was less than 48km, however for respondents in rural areas the travel burden was found to be high, where respondents may have to travel up to 300 kilometres to obtain a mammogram screen. The entire process of getting a subsidised mammogram screen sometimes may involve multiple trips, which can resulted in longer travel distance and higher travel costs compared to a single trip to the screening facility.

In conclusion, availability and spatial accessibility to mammogram screening program in Peninsula Malaysia using the current method of assessment is good in urban areas but is not yet ideal in sub-urban and rural areas. This is a preliminary finding and this study has several limitations. Nonetheless, availability and spatial accessibility issues in mammogram screening need to be addressed in preparation for universal health coverage of breast cancer management in this country.
